# Analgesic effects of Phα1β toxin: a review of mechanisms of action involving pain pathways

**DOI:** 10.1590/1678-9199-JVATITD-2021-0001

**Published:** 2021-11-22

**Authors:** Juliana Figueira da Silva, Nancy Scardua Binda, Elizete Maria Rita Pereira, Mário Sérgio Lima de Lavor, Luciene Bruno Vieira, Alessandra Hubner de Souza, Flávia Karine Rigo, Hèlia Tenza Ferrer, Célio José de Castro, Juliano Ferreira, Marcus Vinicius Gomez

**Affiliations:** 1 Laboratory of Pharmacology, Department of Pharmacy, Federal University of Ouro Preto, Ouro Preto, MG, Brazil.; 2 Graduate Program in Health Sciences, Institute of Education and Research, Santa Casa de Belo Horizonte, Belo Horizonte, MG, Brazil.; 3 Graduate Program in Animal Sciences, State University of Santa Cruz (UESC), Ilhéus, BA, Brazil.; 4 Department of Pharmacology, Institute of Biological Sciences (ICB), Federal University of Minas Gerais (UFMG), Belo Horizonte, MG, Brazil.; 5 Graduate Program in Health Sciences, University of the Extreme South of Santa Catarina (UNESC), Criciúma, SC, Brazil.; 6 Center of Technology in Molecular Medicine, School of Medicine, Federal University of Minas Gerais (UFMG), Belo Horizonte, MG, Brazil.; 7 Department of Pharmacology, Federal University of Santa Catarina, Florianópolis, SC, Brazil.

**Keywords:** Pain, Analgesia, Phα1β peptide, CTK 01512-2, Voltage-activated calcium channels, TRPA1

## Abstract

Phα1β is a neurotoxin purified from spider venom that acts as a high-voltage-activated (HVA) calcium channel blocker. This spider peptide has shown a high selectivity for N-type HVA calcium channels (NVACC) and an analgesic effect in several animal models of pain. Its activity was associated with a reduction in calcium transients, glutamate release, and reactive oxygen species production from the spinal cord tissue and dorsal ganglia root (DRG) in rats and mice. It has been reported that intrathecal (i.t.) administration of Phα1β to treat chronic pain reverted opioid tolerance with a safer profile than *ω*-conotoxin MVIIA, a highly selective NVACC blocker. Following a recent development of recombinant Phα1β (CTK 01512-2), a new molecular target, TRPA1, the structural arrangement of disulphide bridges, and an effect on glial plasticity have been identified. CTK 01512-2 reproduced the antinociceptive effects of the native toxin not only after the intrathecal but also after the intravenous administration. Herein, we review the Phα1β antinociceptive activity in the most relevant pain models and its mechanisms of action, highlighting the impact of CTK 01512-2 synthesis and its potential for multimodal analgesia.

## Background

Pain is an unpleasant sensory and emotional experience associated with actual or potential tissue damage as described by the International Association for the Study of Pain (IASP). It can serve as an index of the severity and activity of a disease condition, a prognostic indicator, and a criterion of treatment efficacy [1]. Chronic pain has an undeniable impact on a patient’s quality of life, with possible financial consequences. Institutional costs associated with chronic pain at a major city university health network hospital in Canada have been estimated to range between CAN$2.5 million and CAN$4.1 million yearly [2].

Neuropathic pain (NP), an example of chronic pathological pain, is complex to manage [3]. NP can be moderated with a wide range of medicines such as tricyclic antidepressants, serotonin-noradrenaline reuptake inhibitors, and calcium-channel-acting modulators (pregabalin and gabapentin) [4]. Ziconotide (Prialt®; Elan Pharmaceuticals, San Diego, CA, USA), a synthetic version of *ω*-conotoxin MVIIA, the Ca_V_2.2 channel blocker, was introduced for the treatment of severe chronic pain that was not relieved by systemic analgesics, adjunctive therapies, or intrathecal morphine [5-8]. Although effective, ziconotide has limited use because of the requirement for i.t. administration coupled with serious neurological and psychiatric adverse events [9].

Studies on *Phoneutria nigriventer*venom showed that Phα1β toxin could inhibit high-voltage-activated (HVA) calcium channel currents and was more potent and effective in inhibiting Ca_V_2.2 channels - N-type voltage-activated calcium channels (NVACC) currents [10]. Phα1β has been shown in many relevant pain models to affect three different types of pain: nociceptive, inflammatory, and pathological [11]. The spider peptide was effective and safe in all tested rodent nociception models [11]. Phα1β demonstrated an extensive analgesic effect with fewer side effects than ω-conotoxin MVIIA, explained by its blockade of HVA calcium channels. Further studies found that Phα1β is an antagonist of the TRPA1 receptor that is also involved in the nociceptive process [12]. The antinociceptive and adverse effects produced by the native toxin form were fully mimicked by its recombinant version, CTK 01512-2, in several pain models [13]. This review focuses on the mechanisms related to the analgesic effect and safety profile of native Phα1β and its recombinant form.

## Phα1β toxin effects in most relevant animal pain models

Experiments on pain using human subjects are ethically limiting, subjective, and practically challenging. Hence, animal models of pain are extensively used to study inflammatory or pathological pain, but the use of animals also possesses ethical constraints and challenges [14]. Phα1β and recombinant CTK 01512-2 have been extensively studied in a wide range of rodent pain models (Table 1). This review focuses on persistent pathological pain models - cancer pain and neuropathic pain (NP) because these pain states are particularly challenging and can be effectively controlled by spider toxins. 

The hot plate or tail-flick test represents models of acute thermal pain where no tissue injury occurs. Souza et al. [15] showed that i.t. delivery of Phα1β (200 pmol/site) produced a long-lasting (3 to 24 h after injection) antinociceptive action in the hot-plate test. The low potency of spider peptides in acute thermal tests [15,16] can be considered a desirable effect that reflects the safety of the toxin. Acute thermal pain, as a nociceptive state, has an important physiological protective function in the preservation of living organisms, and its blockage should be avoided in some circumstances [11].

The formalin test is a preclinical test commonly used to track new compounds with analgesic potential [17-21]. Nociceptive behaviour triggered by formalin injection induces a biphasic behavioural response with a well-defined transition from acute pain to a more persistent pain state [21]. 

The effects of intrathecal administration of the toxin Phα1β on visceral pain (VP) induced by intraperitoneal (i.p.) injection of acetic acid, intracolonic administration of capsaicin, and cyclophosphamide (CPA)-induced haemorrhagic cystitis (HC) have been examined [22,23]. The examination of VP that is the most frequent type of pathological pain remains a challenge for physicians [24-28]. VP animal models have been associated with increases in TRPV1 expression [28-31], a decrease in voltage-sensitive potassium currents, and enhanced expression and function of voltage-sensitive calcium currents [30,31]. Phα1β (50 pmol/site) i.t. pre-treatment inhibited the VP writhes induced by acetic acid or intracolonic behaviours evoked by capsaicin administration [22]. Phα1β (50 pmol/site) displayed significant inhibitory effects on HC-related nociception [23], demonstrating its analgesic potential in visceral pain management.

Incisional surgery in rats and mice produces a sensitive, reproducible, and quantifiable animal model of postoperative pain [32] that is similar to human postoperative pain syndrome in which the surgical incision causes mechanical allodynia and other pain behaviours [33,34]. Intrathecal injection of Phα1β reduced pain indicating behaviours in a mouse model of incisional pain when administered pre- or postoperatively [35,36]. Long-term antinociceptive action suggests that this toxin could also be a therapeutic agent for the control of persistent pain [37]. Numerous results [15,22,23,35,36,37] suggest that spider toxin has the potential to be an efficient and safe alternative for the treatment of various nociceptive and inflammatory pain modalities.

**Table 1.** Analgesic-like effects of Phα1β, CTK 01512-2 and *ω*-conotoxin MVIIA (Ziconotide, Prialt®) in different models of rodent pain.

Models of pain	Peptide toxin		
	Phα1β	CTK 01512-2	*ω*-Conotoxin MVIIA^*^
Nociceptive			
1. Acute spontaneous nociception (irritant agents)^a;b^	+	+	+
2. Heat^c^	+	NT	+
3. Cold^d^	NT	NT	NT
4. Mechanical^e^	+	NT	+
Inflammatory			
1. Irritant-trigged^b^	+	+	+
2. Arthritic^f^	+	NT	+
3. Post-operative	+	NT	+
Neuropathic			
1. Traumatic^g^	+	+	+
2. Nerve differentiation	NT	+	+
3. Chemotherapeutic-agents^h^	+	+	+/-
4. Diabetes-induced^i^	+	NT	+
Visceral pain			
1. Hemorrhagic cystitis^j^	+	NT	+
2. Intracolonic application of agents^b^3. Pancreatitis^l^	+ +	NT +	+ +
Dysfunctional			
1. Fibromyalgia^m^	+	NT	NT
2. Complex regional pain syndrome type 1^n^	NT	+	+
Others			
1. Orofacial pain^a;b;f;g^	NT	+	+
2. Cancer melanoma^o^	+	+	+
3. Opioid-induced	+	NT	NT
4. Multiple sclerosis^p^	NT	+	+

a Formalin; ^b^capsaicin; ^c^hot plate; ^d^acetone or tetrafluoroethene; ^e^Von Frey filaments; ^f^Freund’s complete adjuvant-induced inflammation; ^g^partial sciatic nerve ligation or chronic constriction injury; ^h^paclitaxel or bortezomib; ^i^streptozotocin‑induced diabetes; ^j^cyclophosphamide; ^k^acetic acid; ^l^caused by 5 times hourly cerulein treatment; ^m^caused by repeated reserpine treatment; ^n^exposure to prolonged hind paw ischemia and reperfusion; ^o^B16F10 murine melanoma cells; ^p^myelin oligodendrocytes glycoprotein (MOG_35-55_.)- induced. ^*^Included as positive control; NT: not tested; +: effect; +/-: ω-Conotoxin MVIIA presented effect in chemotherapy-induced neuropathic pain induced by paclitaxel but not in bortezomib, respectively.

### Phα1β antinociceptive effects in a cancer pain model

Cancer-related pain is a prevalent and disabling symptom that requires early prevention and efficient treatment. Currently, opioids are practically the only analgesics capable of controlling severe cancer pain; however, opioid therapy leads to distinct side effects, including the development of analgesic tolerance, sedation, and gut constipation that limit their use [36,38]. Metastatic melanoma is associated with moderate and severe pain, and more than half of these patients require palliative care with morphine therapy [39]. By using an orthotopic tumour inoculation model, Rigo et al. [36] developed a mouse model of skin melanoma that reproduced severe mechanical hyperalgesia in mice. Intrathecal treatment with Phα1β (30 pmol/site) in mice with melanoma remedied this hyperalgesia in a time and dose-dependent manner with an effect that lasted up to 6 h, comparable to the effect of i.t. treatment with *ω*-conotoxin MVIIA [36]. The development of analgesic tolerance is one of the most serious drawbacks of opioids when used repetitively [38]. Using a melanoma model of cancer-related pain in mice, Rigo et al. [36] reproduced an opioid-induced tolerance scenario by administering consecutive doses of morphine for three consecutive days [36]. On the fourth day, the injection of a new challenging dose of morphine was unable to reduce heat hyperalgesia, suggesting analgesic tolerance. Phα1β but not *ω*-conotoxin MVIIA [40], administered 2 h before morphine restored the analgesic effect of this opioid. This suggests that Phα1β could potentially be used as an adjuvant drug for opioid-based cancer pain management. The effect of Phα1β on cancer-related pain in mice was also reproduced with the recombinant form of the toxin [13].

### Phα1β antinociceptive effects in a surgically induced neuropathic pain model

The role of VACC and their inhibitors in neuropathic pain mechanisms has been substantiated [41]. Many surgical animal models such as chronic constriction injury (CCI) of the sciatic nerve, partial sciatic nerve ligation (pSNL), spinal nerve ligation (SNL), spared nerve injury (SNI), brachial plexus avulsion (BPA), sciatic nerve transaction (SNT), and sciatic nerve trisection have been important in the development of chronic pain control. Evidence indicates that the principal pathogenic mechanisms responsible for the induction of neuropathic pain by CCI of the peripheral nerve are associated with oedema, ischemia, macrophage activation (myelin and axonal debris), endoneural extracellular matrix remodelling, cytokine and chemokine upregulation, and other manifestations of neuroinflammation [42-45]. In the pSNL model, i.t. injection of 30 pmol/site of Phα1β caused an anti-allodynic effect from 1 to 4 h after injection and did not alter the normal mechanical sensitivity of the animals [15]. The data from the CCI model showed that administration of Phα1β (200 pmol/site) in the lumbar subarachnoid space blocked the maintenance of mechanical allodynia for up to 4 h after the treatment, with an effect similar to that of *ω*-conotoxin MVIIA [46]. Moreover, other studies demonstrated the anti-allodynic and anti-hyperalgesic effects of Phα1β after a single i.t. injection of 30 or 100 pmol *per site*in a rat model of neuropathic CCI [15,46]. Rats subjected to CCI were implanted with osmotic pumps delivering 60 pmol/μL/h of Phα1β or saline placebo (1.0 μL/h) for 7 days [47]. After the initiation of spinal infusion of Phα1β, a significant antihyperalgesic effect began after 24 h (inhibition of 63% ± 13%) and continued for 6 more days 90% of inhibition on the second day and 100% from day 3 to day 7. Thus, Phα1β attenuated mechanical allodynia in the pSNL and CCI models because of decreased calcium influx into injured sensory neurons.

### Phα1β antinociceptive effects in a chemotherapy-induced neuropathic pain model

Paclitaxel (a taxane-derived anticancer agent) causes peripheral sensory damage resulting in chemotherapy-induced neuropathic pain (CINP); in some patients, an acute pain syndrome appears in the early days of treatment [48]. The mechanism by which chemotherapeutics damage the nervous system and cause CINP is multifactorial and involves inhibition of tubulin dynamics that hampers axonal transport and can lead to axonopathy, loss of epidermal innervation [49,50], oxidative stress, mitochondrial damage [51-54], altered ion channel activity [48,55,56], apoptosis [52], DNA and myelin sheath damage, immunological processes, and neuroinflammation [53,57,58]. The dysregulation of calcium homeostasis has been implicated in the causation of neuropathic pain [58-61]. 

In a model of paclitaxel-induced acute and chronic pain, Rigo et al. [37] evaluated the analgesic potential of two NVACC blockers, *ω*-conotoxin MVIIA and Phα1β. The spider toxin showed a superior therapeutic window compared to the *ω*-conotoxin MVIIA. Phα1β reduced acute and chronic mechanical hyperalgesia induced by paclitaxel and prevented the worsening of the associated chronic pain. Therefore, VACC blockers such as Phα1β reduce synaptic excitation at the level of the spinal cord and could be helpful in the treatment of paclitaxel-induced CINP. TRPA1 expressed in sensory neurons has been shown to contribute to paclitaxel-induced neuropathic pain [62,63]. Phα1β selectively inhibits calcium influx and currents evoked by the TRPA1 agonist on hTRPA1-HEK293, IMR90 fibroblasts, and DRG neurons [12]. The mechanisms involved in the modulation of TRPA1 channels may contribute significantly to acute and chronic cold allodynia and hyperalgesia induced by paclitaxel.

### Phα1β antinociceptive effects in diabetic neuropathic pain model

Diabetic neuropathy (DN) is the most prevalent chronic complication of diabetes [64]. DN is primarily a disorder of sensory nerves; early in the course of DN, patients commonly experience positive sensory symptoms in the feet such as pain, tingling, and paraesthesia, and negative symptoms such as numbness. Disordered sensory processing may evoke allodynia and hyperalgesia [65]. The pathogenesis of DN is multifactorial, and the mechanisms contributing to diabetic DN are not completely understood [66]. It has been suggested that approximately 50% of adults with diabetes are affected by peripheral neuropathy throughout their lifetime [67]. DN induces upregulation of TNF-α and CXCR4 in the DRG at both the early and late phases of DN.

Phα1β, *ω*-conotoxin MVIIA, and AMD3100 (a selective antagonist of CXCR4) administered intrathecally 2 h after STZ-induced DN reduced hypersensitivity in diabetic rats and decreased calcium influx and IL-6 level in the spinal cord [68]. In naïve rats with CXCR4/SDF-1 activation, the induced hypersensitivity decreased after 2 h of treatment with Phα1β or AMD-3100, while *ω*-conotoxin MVIIA did not affect i.t. [68]. The inhibitory effect of Phα1β toxin on diabetic neuropathic pain may involve the CXCR4 chemokine receptor in the spinal cord [68].

## Phα1β and ziconotide toxin safety profile

Ziconotide (*ω*-conotoxin MVIIA) has been approved by the FDA for pain control. However, ziconotide has a narrow therapeutic window, producing maximal analgesia at doses close to the toxic dose, and causing severe side effects that limit its clinical use [69,70,71]. The DT_50_of *ω*-conotoxin MVIIA (ziconotide) is 287 (147-562) pmol/site and for Phα1β is 787 (485-1278) pmol/site [15]. It is noteworthy that Phα1β can produce maximal analgesia at doses that do not induce potential side effects. In contrast, the maximal analgesia induced by *ω*-conotoxin MVIIA (ziconotide) could only be observed at doses close to DT_50_, causing severe side effects [15]. The therapeutic window (DI_50_//DT_50_) for Phα1β and *ω*-conotoxin MVIIA are 16 and 4, respectively [15]. The higher therapeutic window for Phα1β can be explained by several factors including binding to other types of VACC [10] and inhibition of cation channels such as TRPA1 receptors involved in several nociception processes [12].

Miljanich and Ramachandran [72] showed that intrathecal NVACC blockers such as ziconotide (a chemically synthesised version of *Conus magus ω*-conotoxin MVIIA) induce clinical and behavioural effects (shaking behaviour, ataxia, and hyperreactivity) in the central nervous system (CNS) of rats, dogs, and monkeys. Similarly, clinical studies have reported several adverse effects caused by i.t. administration in humans including abnormal gait, ataxia, hypertonia, and tremor [73], with one of the main adverse effects being hypotension [70]. The intravenous (i.v). administration of ziconotide in rats and rabbits has been shown to cause hypotension and increased heart rate (HR) by a combination of sympathetic neurotransmission blockage and mast cell degranulation [74,75]. Currently, ziconotide is administered clinically by a continuous i.t. infusion in the therapeutic management of neuropathic pain, producing a marked analgesic effect in this difficult-to-treat condition [76-78]. Unfortunately, even at analgesic therapeutic doses, ziconotide causes serious side effects [9].

It has been demonstrated that Phα1β inhibits high voltage-activated calcium channels such as NVACC [10]. Our research group studied the possibility that i.t. Phα1β might cause cerebellar-related motor alterations since i.t. injection of N- and P-type calcium channel inhibitors in rats caused the serpentine tail movements and whole-body shaking [79]. After confirming its analgesic potential and safety compared with*ω*-conotoxin MVIIA, the next step was an extensive evaluation of the cardiovascular profile and overall neurological behaviour. The N-type calcium channel is a target for chronic and neuropathic pain [80]. The safety profile of i.t. Phα1β in relevant states of chronic pain has been assessed [15,36,37] as well as the toxic effects of the native peptide after a single or continuous i.t. infusion in a rat model of neuropathic pain [47]. Recently, clinical signs, serum biochemistry, organ weight, and histopathological alterations were evaluated in male and/or female Wistar rats by searching for possible alterations caused by acute i.t. administration of Phα1β at a high dose [81]. Phα1β i.t. injection produced maximum analgesia after doses (100-200 pmol/site) that did not induce the described potential side effects, with a therapeutic window of 16 [15]. Only dynamic allodynia was observed in an intrathecally delivered dose of 100 pmol [13]. In comparison, the maximal analgesia induced by *ω*-conotoxin MVIIA (100 pmol/site) could only be observed in doses that cause severe side effects with a therapeutic window of 4 [15].

The pre-clinical tests performed to establish a cardiovascular profile and overall neurological behaviour showed that i.t. Phα1β (200 pmol/site) did not change the mean arterial blood pressure or HR 3 h after the injection. However, i.t. *ω*-conotoxin MVIIA (100 pmol/site) induced an increase in HR 3 h after administration [35]. Treatment with the toxin did not alter neurological performance after 3 h, suggesting the absence of causing neurological deficits in rats [35]. Even in a paclitaxel-induced acute and chronic pain model, i.t. *ω*-conotoxin MVIIA (10-100 pmol/site) caused adverse effects while Phα1β (30-300 pmol/site) produced only minor adverse effects when injected at the acute or chronic pain stage [37]. The same results were reproduced in a cancer-related pain model; *ω*-conotoxin MVIIA showed adverse effects (such as sedation, motor dysfunction, and paradoxical hyperalgesia) at all tested doses, while Phα1β produced minimal adverse effects (paradoxical hyperalgesia) only at the highest tested dose [37]. 

Continuous intrathecal infusion of an NVACC blocker is a critical option for neuropathic pain management [80]. The Phα1β’s antinociceptive and toxic effects were compared after a single continuous i.t. infusion in a rat model of NP induced by CCL of the sciatic nerve. A single injection of Phα1β (30 or 100 pmol/site) or continuous infusion (60 pmol/μL/h for 7 days) was able to reverse nerve injury-induced nociception [47]. In both forms of administration, the toxin did not cause behavioural side effects or histopathological changes in the CNS. In a single or continuous injection, intrathecal administration of ziconotide causes nausea, confusion, postural hypotension, allodynia, abnormal gait, urinary retention, and weakness, and severe side effects that tend to occur more commonly at higher doses [73-78]. The detailed alterations related to the behavioural side effects are described in Table 2.

Dellagrave et al. [81] evaluated clinical signs, relative organ weight, biochemical parameters, and histopathological alterations in hepatic and renal tissues. Clinical signs manifested by Phα1β (500 pmol/site) injected in male rats only showed dyspnoea, while females manifested decreased touch response and tremors. There were no significant differences in the weights of the male and female organs. Serum biochemical data in male rats revealed a significant reduction within the physiological limits of species related to urea, AST, ALT, ALP, and hepatic and renal congestion [81]. Evaluation of the potential cytotoxic, genotoxic, and mutagenic effects of Phα1β by different methods showed that Phα1β (500 pmol/site) induced DNA damage in the spinal cord but not in peripheral blood [82]. In conclusion, the native toxin showed a good safety profile with transient signs of clinical toxicity [81] and genotoxic effects only in SNC [82] at doses five times higher than those used to obtain the analgesic effect. The results demonstrate that Phα1β produces analgesia after single or continuous i.t. delivery in relevant models of acute and chronic pain eliciting minimal toxic effects and with a greater therapeutic window of 16, higher than that 4 of *ω*-conotoxin MVIIA [15].

**Table 2.** Side effects of Phα1β, CTK 01512-2 and *ω*-conotoxin MVIIA (Ziconotide, Prialt®) in different doses or administration routes.

	Peptide toxin	Phα1β						CTK 01512-2						*ω*-Conotoxin MVIIA^*^		
	Routes	Intrathecal route					Intrathecal continuous infusion	Intrathecal route			Intravenous route			Intrathecal route		
	Doses	10 pmol/site	30 pmol/site	100 pmol/site	200 pmol/site	300 pmol/site	60 pmol/ul/h	30 pmol/site	100 pmol/site	200 pmol/site	0.2 mg/kg	0.6 mg/kg	1.8 mg/kg	10 pmol/site	30 pmol/site	100 pmol/site
**Adverse effects and related parameters**	**Serpentine tail**	Absent^r^	Absent^r^	Absent^rm^	Absent^m^	Absent^r^	Absent^r^	Absent^m^	Absent^m^	Not tested	Absent^m^	Absent^m^	Absent^m^	Present^rm^	Absent^rm^	Absent^rm^
	**Body shake**	Absent^r^	Absent^r^	Absent^rm^	Absent^m^	Absent^r^	Absent^r^	Absent^m^	Absent^m^	Not tested	Absent^m^	Absent^m^	Absent^m^	Present^rm^	Absent^rm^	Present^rm^
	**Dynamic allodynia**	Absent^r^	Absent^r^	Present^rm^	Absent^m^	Absent^r^	Absent^r^	Absent^m^	Present^m^	Not tested	Absent^m^	Absent^m^	Absent^m^	Present^rm^	Present^rm^	Present^rm^
	**Sedation**	Not tested	Absent^m^	Absent^m^	Not tested	Not tested	Not tested	Not tested	Not tested	Not tested	Not tested	Not tested	Not tested	Not tested	Present^rm^	Present^rm^
	^**^Forced motor activity impairment	Not tested	Not tested	Absent^m^	Absent^m^	Not tested	Not tested	Not tested	Absent^m^	Not tested	Absent^m^	Not tested	Not tested	Absent^m^	Not tested	Not tested
	^***^General motor activity impairment	Not tested	Not tested	Absent^m^	Absent^rm^	Not tested	Not tested	Not tested	Absent^m^	Absent^r^	Not tested	Not tested	Not tested	Absent^m^	Not tested	Absent^r^
	**Mean arterial pressure**	Not tested	Not tested	Not tested	Unaffected^r^	Not tested	Not tested	Not tested	Not tested	Not tested	Unaffected^m^	Unaffected^m^	Unaffected^m^	Not tested	Not tested	Not tested
	**Heart frequency**	Not tested	Not tested	Not tested	Unaffected^r^	Not tested	Not tested	Not tested	Not tested	Not tested	Unaffected^m^	Unaffected^m^	Unaffected^m^	Not tested	Not tested	Not tested

r Rats; ^m^mice. ^*^Included as a positive control for Phα1β and CTK 01512-2- studies from other groups were not considered; ^**^evaluated by the Rotarod method; ^***^evaluated by the open field method.

## Phα1β toxin action mechanisms

Phα1β toxin has been proven to inhibit HVA calcium channels and act as a TRPA1 antagonist. This inhibitory effect is most useful in controlling pain due to the overexpression or increased activity of the molecular agents in these disease conditions. Spider peptide activity on the nervous system has been extensively investigated through events related to high-voltage activated calcium channels (HVACC) and TRPs such as intracellular calcium transients, neurotransmitter release, oxidative stress pathways, and inflammatory mediators (Table 3). This review focuses on the effects of Phα1β on molecular targets, calcium influx, glutamate release, and reactive oxygen species (ROS) generation as the most important and described mechanisms related to pain pathways. Glial plasticity effects have also been reported and are detailed in Table 3.

**Table 3:** Phα1β, CTK 01512-2 and *ω*-conotoxin MVIIA (Ziconotide, Prialt®) pain pathway action mechanisms.

Action mechanisms	Peptide toxin		
	Phα1β	CTK 01512-2	*ω*-Conotoxin MVIIA^*^
Molecular targets	IC_50_(nM)		
Voltage gated calcium channel^a^			
1. N- type VACC	122	Not tested	Not tested
2. R- type VACC	136	Not tested	Not tested
3. P/Q - type VACC	263	Not tested	Not tested
4. L - type VACC	607	Not tested	Not tested
5. T - type VACC	Not tested	Not tested	Not tested
Transient receptor potential^a;b;c^			
1. TRPV1	Unaffected	Unaffected	Not tested
2. TRPV4	Unaffected	Unaffected	Not tested
3. TRPA1	681^a^;40^b^;32^c^	506^a^;28^b^;34^c^	Not tested
**Molecular targets related events**			
Intracellular Ca^2+^	Decrease^c;d;e^	Decrease^c;d;e^	Decrease^c;e^
Glutamate release	Decrease^e;f^	Decrease^f;g^	Decrease^e;f;g^
Oxidative stress			
3.1 ROS generation	Decrease^f^	Not tested	Decrease^f^
3.2 Lipid peroxidation	Not tested	Decrease^c^	Decrease^c^
3.3 Myeloperoxidase activity	Decrease^e;h^	Not tested	Unaffected^e;h^
3.4 Malondialdehyde levels	Decrease^e^	Not tested	Not tested
Inflammatory mediators			
4.1 TNF-α	Decrease^i^	Decrease^e;h^	unaffected^;h;i^
4.2 IL-1β	Decrease^i^	Decrease^e;h^	Decrease^e;h;i^
4.3 IL-6	Decrease^a^	Not tested	Decrease^a^
4.4 IL-10	Increase^i^	Increase^e;h^	Unaffected^e;h;i^
Glial plasticity^e^			
5.1 GFAP	Decrease	Decrease	Decrease
5.2 Iba-1	Unaffected	Unaffected	Unaffected
5.3 Microglia proliferation	Decrease	Not tested	Not tested
5.4 Astrocyte proliferation	Decrease	Not tested	Not tested

a Human embryonic kidney (HEK) 293 cells and N18 neuroblastoma cells; ^b^IMR90 cells; ^c^DRG neurons; ^d^TRPA1-HEK293; ^e^spinal cord samples; ^f^CSF; ^g^trigeminal ganglia; ^h^brain tissue; ^i^bladder, ^j^paw skin. ^*^Included as a positive control for Phα1β and CTK 01512-2 studies from other groups were not considered. Note: VACCs are shown in order of preference for Phα1β.

### High voltage-activated calcium channel blockade by Phα1β toxin

The activity of HVACC in different types of pain derives from their heterogeneity in structure, and tissue and cell localisation [83]. The calcium channel family consists of different channel subtypes that can be divided based on the voltage dependence of activation: HVA calcium channels into L-type (Ca_V_1.1-Cav1.4), P/Q-type (Ca_V_2.1), N-type (Ca_V_2.2), R-type (Ca_V_2.3), and low-voltage activated channels, T-type (Ca_V_3.1, Ca_V_3.2, Ca_V_3.3) [84]. There is literature evidence implicating low-voltage calcium channel in pain pathologies [84] and Phα1β was no tested on the low-voltage activated channels. The NVACC are almost exclusively expressed in neuronal tissue and localised in synaptic nerve terminals in laminae 1 and 2 of the dorsal horn, where their opening results in the release of neurotransmitters such as CGRP, glutamate, and substance P [84,85]. Consequently, inhibiting calcium influx in the Ca_V_2.2 channel results in reduced neurotransmission and analgesia. Therefore, these calcium entry pathways are targets for therapeutic agents in the treatment of disorders such as pain management [86]. 

Vieira et al. [87] demonstrated that Phα1β inhibits calcium influx and decreases glutamate Ca^2+^-dependent exocytosis from cortical synaptosomes, suggesting that the toxin targets calcium channels. Electrophysiological recordings show that Phα1β blocks mammalian calcium ion currents in HVA calcium channels exogenously expressed in HEK cells [10]. Four HVA calcium channels were examined in this study; the blockade by Phα1β was the most potent and effective on Ca_V_2.2 (N-type voltage-activated calcium channels), blocking > 95%. In addition to the blockade of Cav 2.2 channel, Phα1β partially reduced the conductance of Ca_V_1 (L-type), Ca_V_2.1 (P/Q-type), and Ca_V_2.3 subtypes (R-type). The suggested mechanism of action of Phα1β in calcium channel blockade is the complete blockade of Ca_V_2.2 currents. It seems that the native peptide may bind tightly to the external mouth of the channel and physically occlude the pores. When Phα1β action on Cav1, Cav2.1, and Cav2.3 subtypes was evaluated, an incomplete blockade was observed, suggesting that the Phα1β effect might be associated with a state-dependent affinity between the channel and the toxin [10]. Literature reports that several blockers of voltage-activated Ca^2+^channels exhibit state and/or potential-dependent blockage [88-89]. However, Phα1β was tested at concentrations up to 1 µM; thus, higher concentration of the toxin may achieve the complete blockage of these channels. The order of potency of Phα1β inhibition on calcium currents was N-(a1B/Cav2.2) > R-(a1E/Cav2.3) > P/Q-(a1A/Cav2.1) > L-(a1C/Cav1.2) [10]. Therefore, Phα1β exhibited a measurable preference for Ca_V_2.2 calcium channel, with the blockade being reversible. These results showed that blockade of NVACCs has pharmacological utility in the management of pain.

### TRPA1 channel antagonism by Phα1β

**Figure 1.**
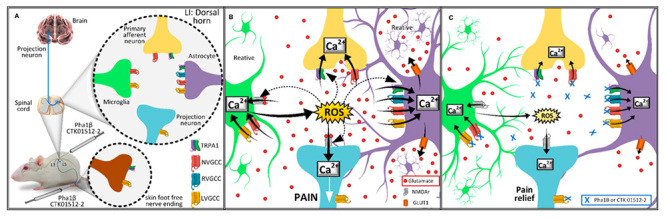
Molecular targets and action mechanisms involved in the intrathecal injection of Phα1β and CTK 01512-2 peptide. **(A)**Molecular targets of Phα1β and CTK 01512-2 and their cellular localization on the periphery tissue and lamina I of the spinal cord. **(B)**Phα1β and CTK 01512-2’s molecular target activation related events in spinal cord lamina I during pain states. **(C)**The suggested mechanism for pain relief through molecular targets blockade by Phα1β and CTK 01512-2. Note: more studies are necessary to understand how native peptides and their recombinant versions interact with HVCACC and TRPA1.

TRPA1 is a nonselective cation channel expressed in nociceptive somatosensory neurons of the DRG, trigeminal, and nodose sensory ganglia, acting as a cellular sensor to several harmful physical and chemical stimuli [90-91]. This channel is a member of a subset of transient receptor potential (TRP) channels subdivided into seven main subfamilies according to their homology and channel function: TRPC (canonical), TRPV (vanilloid), TRPM (melastatin), TRPML (mucolipin), TRPP (polycystin), TRPA (ankyrin transmembrane protein), and TRPN (Nom PC-like) [92]. This receptor can be activated and modulated by endogenous agonists derived from inflammatory or tissue injury conditions, thus contributing decisively to the pathogenesis of inflammation and pain, possibly in the transition from acute to chronic pain [92-93]. Studies involving the TRPA1 receptor have been carried out to develop new therapeutic tools for the treatment of pain. Tonello et al. [12] demonstrated that Phα1β inhibits HC-030031 (a TRPA1 receptor antagonist) and currents evoked by TRPA1 channel stimulation in HEK293 cell cultures (Figure 1). Phα1β reduced nocifensive responses evoked by allyl isothiocyanate, a TRPA1 agonist, by intraplantar and i.t. administration, attenuating mechanical and cold hyperalgesia in a model of NP pain induced by bortezomib. This study also showed that the recombinant peptide did not exert action on other TRP channels such as TRPV1 and TRPV4, suggesting its selectivity by the TRPA1 channel [12]. Previous findings have demonstrated that Phα1β does not inhibit the TRPV1 channel, corroborating the fact that this toxin does not affect other TRP channels [94]. 

### Reduced glutamate release by Phα1β toxin

N-type calcium channels are preferentially coupled to glutamate release in the enhanced nociceptive transmission at the spinal level following formalin inflammation [95]. Phα1β and *ω*-conotoxin MVIIA blocked glutamate release evoked by capsaicin in isolated nerve terminals from the spinal cord, but Phα1β’s potency was about two times greater than that of *ω*-conotoxin MVIIA [15]. The IC_50_for the inhibitory effect on glutamate release on the nerve terminal by Phα1β was 2.1 µmol while for *ω*-conotoxin MVIIA it was 4.8 µmol [15]. It is noteworthy that different pain models increase Glu levels in the cerebrospinal fluis (CSF) [15,95-98]. The antinociceptive and adverse effects produced by the native toxin form were fully mimicked by the CTK 01512-2 recombinant version in several pain models [13] (Figure 1). Moreover, in isolated nerve terminals obtained from the spinal cord, the spider toxin also blocked Glu release evoked by capsaicin [15]. Vieira et al. [87] demonstrated that Phα1β inhibits calcium influx and decreases glutamate Ca^2+^-dependent exocytosis from cortical synaptosomes, suggesting that the toxin targets calcium channels. We believe that a reduction in excitatory neurotransmitter release from presynaptic terminals by decreasing calcium influx would lessen the activity of the dorsal horn neurons and thus raise the threshold for nociceptive response.

### Reduced reactive oxygen species generation by Phα1β toxin

Several studies have demonstrated that increased intracellular ROS, reactive nitrogen species (RNS), and Ca^2+^play a major role in the aetiology of pain processes [99,100]. Interactions between ROS and calcium signalling can be considered as bidirectional, wherein ROS can regulate cellular calcium signalling, while calcium signalling is essential for ROS production [101]. Therefore, the elevation of intracellular calcium levels is responsible for the activation of ROS-generating cytosolic enzymes and the formation of free radicals by the mitochondrial respiratory chain. In contrast, ROS can significantly affect calcium influx into cells and intracellular calcium stores [102]. 

Some studies have reported that excessive ROS and RNS production in rat models involves TRPA1 channels in the aetiology of pain processes [103]. The cellular mechanisms have not been fully clarified, although there are some reports on TRPA1 activation-induced pain processes such as diabetic peripheral pain [104,105], spinal cord injury-induced pain [106,107], and chemotherapeutic agent-induced pain [108]. Furthermore, sodium channel blockers reduce the influx of calcium into the cells, thereby reducing the production of free radicals and attenuating lipid peroxidation reactions [109]. This evidence suggests that this crosstalk between calcium influx and ROS/RNS generation plays an essential role in many pathophysiological conditions including neurodegenerative diseases such as Parkinson’s, Alzheimer’s, and inflammatory diseases [101], and neuropathic pain [110]. 

The effects of Phα1β on the generation of ROS and proinflammatory mediators have been observed in pain models [22,23] (Figure 1). In the VP intracolonic capsaicin model, *ω*-conotoxin MVIIA attenuation of depolarization-induced Ca^2+^influx, specifically in NVACC, was less effective than Phα1β in reducing ROS levels [22]. The higher effect of Phα1β is most likely due to its HVA calcium channel current inhibition [10] and TRPA1 channel blockade [12]. The marked analgesic, anti-inflammatory, and recovery of functional actions promoted by Phα1β appear to rely on the reduction of neutrophil migration that in turn might reduce oxidative stress.

### Glial structural plasticity reversal by Phα1β toxin

The pain process and glial activation are directly related [111,112]. Proinflammatory molecules released at the injury site can stimulate sensory neurons in the peripheral terminal and release several pro-algesic substances [113]. We found that CFA-induced hind paw inflammation in rats produced robust morphological changes in spinal astrocytes and microglia, compatible with the reactive phenotype [114]. These glial changes include an increase in GFAP protein expression in astrocytes [115-117] and Iba1 or OX-42 proteins in microglia [118-121].

In addition to its analgesic properties, the Phα1β spider toxin reverses glial changes caused by peripheral inflammation [115], reducing the overexpression of GFAP and Iba1 in short-time astrogliosis (2 days) and long-term microgliosis (14 days). These effects were more apparent in rats treated with the Phαβ spider toxin than with ω-conotoxin MVIIA, a specific N-type calcium channel antagonist. Microglia proliferation induced by CFA peripheral inflammation was not observed. Intriguingly, treatment with *ω*-conotoxin MVIIA toxin produced a significant increase in microglia proliferation [115]. Microglial cells express a myriad of receptors such as calcium channels [122,123]. Glial plasticity depends on intracellular and extracellular calcium signalling which is important for regulating glial autocrine signalling, structural plasticity, and proliferation [124,125]. Phα1β might exert an effect on glial calcium channels because of its ability to act as a VACC inhibitor. However, it is still unclear whether Phα1β toxin acts directly or indirectly in glial cells. 

## Recombinant CTK 01512-2 shows effects similar to the native Phαβ toxin

The development of the recombinant version of Phα1β named CTK 01512-2 arose because of the difficulty of getting significant amounts of spider venom and obtaining the native toxin by purifying spider venom. Giotto Biotech S.r.l. (Florence, Italy) synthesised this recombinant form through expression in *Escherichia coli*. The CTK 01512-2 have an identical sequence of the 55 amino acids as the native Phα1β toxin (ACIPRGEICTDDCECCGCDNQCYCPPGSSLGIFKCSCAHANKYFCNRKKEKCKK) and six disulphide bonds [126]. The recombinant peptide showed strong analgesic activity as the native toxin, with negligible side effects [13]. It reduced mechanical hyperalgesia induced by CCl in the sciatic nerve [13]. In a deafferentation pain model, CTK 01512-2 attenuated mechanical allodynia, cold allodynia, and thermal hyperalgesia without affecting the locomotor and exploratory activity of the rats [127]. Orofacial pain is a painful condition that affects the soft and hard tissues of the head, face, and neck [128,129]. CTK 01512-2 reduced orofacial hyperalgesia in the formalin-induced inflammatory phase in the lip and intraarticular CFA injections [130].

The recombinant Phα1β showed a marked antiproliferative effect on glioblastoma cells after i.t. administration blocking NVACC [131], anti-hyperalgesic effects on cancer melanoma cells [36], and inhibition of capsaicin nociceptive behaviour [37]. Intrathecal treatment with recombinant peptides also modulates other events such as neuroinflammation and neurodegeneration [132,133]. In addition to pain signalling, there is evidence that VACC also participate in the development of some CNS disorders. In the model of experimental autoimmune encephalomyelitis (EAE) induced by myelin oligodendrocyte glycoprotein (MOG_35-55_), the recombinant peptide administered i.t. showed antinociceptive activity [132], improving cognitive deficits and motor coordination, modulating the disease progression, and attenuating neuroinflammatory changes with higher efficacy than ziconotide and fingolimod [132]. Notably, i.v CTK 01512-2 attenuated the symptoms of the EAE model, while *ω*-conotoxin MVIIA did not by this administration route [132]. CTK-01512-2 significantly improved the neuroinflammatory response in this model of multiple sclerosis (MS), reducing the levels of TNF, IL-1B, IFN-γ, IL-17, and IL-23 in the brain and spinal cord. These results indicate that the recombinant CTK-01512-2 greatly improved the neuroinflammatory responses with higher efficacy when compared to ziconotide, suggesting that this molecule is a promising adjuvant for MS management. 

Acute pancreatitis (AP) is an inflammatory disease of the pancreas. Agents that modulate the activity of high-voltage activated calcium channels such as Phα1β [10] and *ω*-conotoxin MVIIA [70,78] exhibit experimentally and clinically significant effects by relieving chronic pain in AP. In rodents, i.p. injections of cerulein induces AP as evidenced by an increase in hyperalgesic pain, inflammatory infiltration, amylase and lipase secretion, and reactive oxygen species formation [133]. Phα1β and its recombinant CTK 01512-2 form, both blockers of the TRPA1 receptor [12] and HVACC [10], abolished these effects [133] after i.t. administration. *ω*-Conotoxin MVIIA, a selective inhibitor of N-type HVACC [72], did not affect the induced increase in pancreatic enzyme secretion. Phα1β has been shown to have an antinociceptive effect in several rodent pain models, including visceral pain [22], postsurgical, inflammatory, and neuropathic pain [15,37,47], and cancer pain [36]. Intrathecal treatment with Phα1β and recombinant CTK 01512-2 did not significantly alter the spontaneous locomotion of rats with AP, whereas *ω*-conotoxin MVIIA did affect it. These results suggest the potential use of Phα1β and recombinant CTK 01512-2 as analgesic drugs for the treatment of acute pancreatitis.

The analgesic and side effects of i.v. administered CTK 01512-2 were also studied in the CCL-induced neuropathic pain and paclitaxel-induced acute and chronic pain in which the recombinant toxin exerted analgesic action. The analgesic effects were not accompanied by acute toxicity compared to morphine that induced significant changes in motor activity, HR, and blood pressure [134]. The analgesic effect was also elicited in male and female mice by CTK 01512-2 (0.06 and 0.6 mg/kg i.v) in a complex regional pain syndrome 1 model; the peptide attenuated mechanical and cold allodynia in the acute and chronic nociceptive state [135]. 

CTK 01512 2 is a selective antagonist of the TRPA1 channel as its natural toxin [12], producing *in vivo*peripheral and central antinociceptive effects via TRPA1 channel antagonism without affecting other TRP channels such as TRPV1 and TRPV4 [94].

The effect of CTK 01512 2 on glutamate levels, ROS generation, lipid peroxidation, DNA damage, and inflammatory mediators have been observed in pain models. Future studies are required to confirm that the recombinant peptide has potential for clinical use.

## Phα1β and CTK 01512-2 peptides as potential drugs for multimodal analgesia

Studies addressing the analgesic potential of opioids combined with calcium channel blockers are scarce. In terms of opioid addiction, it has been estimated that more than 2 million people suffer from opioid-related substance abuse disorders [136]. The management of pain in opioid-tolerant patients is one of the most challenging aspects, especially when opioids are prescribed for chronic pain or addiction-related opioids. Preoperative use of opioids has been associated with worse surgical outcomes [137]. This is troubling because the use of opioids has steadily increased, and the number of readmitted patients who are tolerant to opioids is 8% [137]. Opioid-sparing multimodal analgesia protocols are a critical component of clinical practice and surgical guidelines [138,139]. Thus, multiple target agents such as native Phα1β and its recombinant version HVA calcium channel blockers and TRPA1 antagonists might be excellent candidates not only for composing a synergistic effect but also perhaps for reversing adverse effects such as tolerance [36]. Repeated morphine treatment causes tolerance, hyperalgesia, withdrawal syndrome, and constipation, but a survey by Tonello et al. [16] showed that Phα1β and CTK 01512-2 were able to reverse these effects. In rats, the ability of Phα1β to restore the analgesic effect of morphine under opioid-tolerance regimens is worth noting, suggesting some *in vivo*interaction of the two drugs when they are used together [36]. Administration of morphine at an ineffective dose (3-10 mg/kg) in the presence of Phα1β or CTK 01512-2 (30 pmol/site) culminates in a better analgesic effect than administering peptides or morphine alone [16]. These data showed that Phα1β and its recombinant version are effective in potentiating analgesia caused by a single dose of morphine as well as in reducing tolerance and the adverse effects induced by repeated administration of morphine, indicating their potential use adjuvant drugs in combination with opioids. Further studies are needed to determine the degree of interactions between the two classes of drugs involved in adverse events. In conclusion, both native Phα1β and CTK 01512-2 have the potential for use by parenteral route multimodal pain therapy as well as in other CNS disorders due to their varied mechanisms of action.

## Conclusions

Studies with Phα1β and recombinant CTK 01512-2 have proven their analgesic profile in nociceptive, inflammatory, and pathological pain through HVACC and TRPA1 inhibition. Events related to molecular targets such as calcium transients, glutamate release, glial plasticity, ROS, and inflammatory mediator production have been described, supporting their antinociceptive effects and safety profiles. This review covers the 15 years of Phα1β research since the identification of the first target by Vieira et al. [10]. Currently, there has been an increase in the number of papers published on native and recombinant Phα1β, stimulated by the availability of the recombinant version. Although further pharmacokinetic and preclinical (including toxicity profile in other species) studies are still necessary, we believe that these peptides are close to being developed as alternative clinical drugs for the severe chronic pain management and multimodal analgesia protocol application.
